# 糖源碳点的制备及其在生物医药与环境污染物分析中的应用

**DOI:** 10.3724/SP.J.1123.2025.02003

**Published:** 2025-08-08

**Authors:** Xuanyu LIU, Lei SUI, Mingyu MA, Yi BI, Zhihua SONG

**Affiliations:** 烟台大学药学院，新型制剂与生物技术药物研究山东省高校协同创新中心，分子药理和药物评价教育部重点实验室，山东 烟台 264005; School of Pharmacy of Yantai University，Collaborative Innovation Center of Advanced Drug Delivery System and Biotech Drugs in Universities of Shandong，Key Laboratory of Molecular Pharmacology and Drug Evaluation，Ministry of Education，Yantai 264005，Shandong

**Keywords:** 碳点, 糖, 合成方法, 药物, 新污染物, 分离分析, carbon dots（CDs）, saccharides, synthesis method, pharmaceuticals, emerging contaminants, separation and analysis

## Abstract

碳点（carbon dots，CDs）是一类应用广泛的新型材料。CDs具有小尺寸效应、显著的光稳定性、较低的细胞毒性、良好的生物相容性、易制备、易于表面修饰和表面功能基团（羟基、羧基、氨基等）丰富等优点，在各个领域表现出超高潜力。糖类化合物是自然界中容易获得的碳水化合物，具有无毒性和低渗透性，为合成具有特殊性质和多功能应用的CDs提供了一种有吸引力且廉价的起始材料。近年来，生物基糖源制备CDs因成本低、原料可再生、绿色环保等优势为CDs合成提供了新思路。已有文献中主要通过“自上而下”和“自下而上”两种方法合成CDs。本文总结了以糖为碳源通过“自下而上”法（如水热法、微波辅助法、超声法、热解法）合成具有高水溶性、低毒性、光稳定性和化学稳定性等优点的CDs。这些CDs在生物成像、生物传感、药物/基因载体、色谱分析等多个领域具有广阔的应用前景。在生物成像方面，CDs具有优异的光学性能和低毒性可实现细胞和组织内实时成像。在生物传感方面，CDs表面官能团的相互作用可实现生物分子/离子的高灵敏度检测。在药物/基因递送方面，CDs可作为高效载体并降低副作用。在色谱分析方面，CDs在固定相上负载可实现化合物的高效分离。此外，CDs在核素、抗生素等新污染物以及生物碱、核苷类化合物等药物分离分析方面展现出很好的性能，为环境监测和药物分析提供了新的工具。未来，CDs的开发应聚焦以下几个方面：开发低成本、大规模化的制备方法；优化CDs表面功能化；开发杂原子改性的CDs；拓展色谱和传感应用并深化作用机理的研究。

## Abstract (en)

Carbon dots（CDs） are a new class of materials with a wide range of applications. CDs possessed the advantages of small size effect， remarkable photostability， low cytotoxicity， good biocompatibility， simple synthesis and surface modification， and abundant surface functional groups（hydroxyl groups， carboxyl groups， amino groups， etc.）. Currently， CDs show ultra-high potential in various fields. Carbohydrates are one of the most diverse and important classes of biomolecules in nature and can be modified at isomeric positions and hydroxyl functional groups. The saccharides are readily available carbohydrates in nature with non-toxicity and low permeability， providing an attractive and inexpensive starting material for the synthesis of CDs with specific properties and multifunctional applications. In recent years， the preparation of CDs from bio-based saccharide sources provides a new idea for CDs synthesis due to its advantages of low cost， renewable raw materials and green environment. Depending on the carbon source， the synthesis strategy of CDs can be broadly classified into top-down and bottom-up approaches. The top-down approach refers to the decomposition of larger carbon structures into nanoscale particles（e.g. graphene， carbon nanotubes， etc.）， while the bottom-up approach refers to the synthesis of CDs from smaller carbon units（e.g. saccharides， organic acids， etc.）. Using saccharides as carbon source， the methods for synthesis of CDs via bottom-up approach have been carefully summarized. In detail， bottom-up approach consisted of hydrothermal， microwave-assisted， ultrasonic and pyrolysis methods. The acquired saccharide-derived CDs had the properties of good water-solubility， low-toxicity， photostable and chemically stability. What’s more， the obtained CDs had broad application prospects in various fields， such as bioimaging， biosensing， drug/gene delivery and chromatographic analysis. In bioimaging， CDs have excellent optical properties and low toxicity， and can be used as fluorescent probes for real-time imaging in cells and tissues. In biosensing， the functional groups on the surface of CDs can specifically bind to the detected substances to achieve highly sensitive detection of biomolecules or ions. In drug/gene delivery， CDs can be used as carriers for efficient delivery of drugs or genes， reducing side effects and improving therapeutic effects. In chromatographic separation， CDs can be loaded on stationary phases and interact with compounds to achieve efficient separation of compounds. Furthermore， the saccharide-derived CDs showed good performances in the separation and analysis of new contaminants including nuclides， antibiotics， etc. Additionally， the saccharide-derived CDs exhibited outstanding properties for pharmaceuticals（alkaloids， nucleoside analogues， etc.） determination. Thus， CDs provide new tools for environmental monitoring and drug analysis. In the future， the research objectives in the preparation of CDs include the following： firstly， to continue to develop low-cost and simple preparation methods for the large-scale production of physically and chemically stable CDs. Secondly， the surface functional groups of CDs are further enriched in order to improve the interaction ability of CDs with target molecules or ions and to be able to endow CDs with more functionalities， such as targeting and responsiveness. In addition， a variety of heteroatoms（e.g.， nitrogen， boron， phosphorus， sulfur， etc.） are doped to improve the properties of CDs. In addition to the improvement of preparation protocols， expanding the application of CDs in chromatography and sensing analysis， as well as in-depth study of their mechanism of action still deserves attention.

近年来，碳纳米材料（包括石墨烯^［1］^、富勒烯^［2］^、碳纳米纤维^［3］^和碳纳米管^［4］^等）受到了研究人员的广泛关注。碳点（carbon dots，CDs）属于碳纳米材料家族，具有独特的荧光特性。早在2004年，Xu等^［5］^在采用电弧放电法净化单壁碳纳米管时，意外发现了*sp* ^2^和*sp* ^3^聚合的CDs。2006年，Sun等^［6］^对该类新材料进行了命名。后来，CDs被定义为精细分散，尺寸小于10 nm，且被羟基、氨基、羧基或芳香环等官能团包裹的碳颗粒。基于其基本组成，主要分为4类：（1）具有二维层状石墨烯核心的石墨烯量子点（graphene quantum dots，GQDs）；（2）具有球形晶体核心的碳量子点（carbon quantum dots，CQDs）；（3）具有非晶态核的碳纳米点（carbon nanodots，CNDs）；（4）具有高度交联聚合物框架和轻微碳化疏水核心的碳化聚合物点（carbonized polymer dots，CPDs）^［7-9］^。CDs除了具有传统半导体量子点的高量子产率和可调节的发射波长外，还表现出优异的光稳定性、较低的细胞毒性、特殊的生物相容性、灵活的表面修饰和显著的化学惰性等优点^［10］^。因此，CDs在细胞成像^［11，12］^、体内成像^［8，13］^、药物/基因载体^［14，15］^、生物传感^［16，17］^、光催化^［18，19］^、色谱分析^［20，21］^等各个领域表现出超高应用潜力。

碳水化合物是自然界中最多样化和最重要的一类生物分子，它提供了定义明确的手性支架，可以在异构位置和羟基官能团上进行修饰。糖是一类无毒性和低渗透性的碳水化合物，与其他前驱体相比具有以下优点：其表面含有丰富的羟基，在碳化的过程中容易生成丰富的羟基、醚键、羧基等，使制备的CDs具有优异的亲水性和生物相容性；部分糖类化合物合成CDs时自然地引入杂原子（如几丁质、壳聚糖、氨基葡萄糖等），通过氢键、*π-π*、偶极-偶极、离子交换及静电相互作用等方式，增强与被测物质的相互作用；此外，由糖作为碳点的前驱体，合成过程中会保留大量手性中心，能够实现部分化合物的立体选择性识别^［22，23］^。糖类化合物表面的基团较为常见，其广谱结合的特性容易产生非特异性吸附，科研人员通过对其表面进一步功能化修饰，例如结构修饰、分子印迹、动态响应设计等，提高其与被测物质特异性结合的能力^［24-27］^。根据目标物的特性，糖源碳点可以通过表面修饰识别标记生物分子、重金属离子、细胞/细胞器、环境污染物等化合物。因此糖源碳点的应用能够系统性覆盖从离子/分子检测到活体检测的多层次应用，其功能化修饰策略有效地克服了表面常见基团的局限性，为靶向治疗和精准检测提供了新思路。因此，糖类化合物为CDs合成提供了一种成本廉价、来源广泛的理想前驱体。目前，应用最广泛的是单糖及其衍生物，例如葡萄糖、糖胺、甘露糖、果糖等，以及二糖，如蔗糖、乳糖和麦芽糖等，已被用于使用不同的方法制备CDs^［22，28］^。在天然生物聚合物领域，储量丰富的多糖如纤维素、淀粉、壳聚糖、藻酸盐等在CDs合成方面，同样展现出优异的合成潜力^［28-30］^。近年来，生物基糖源由于其具有成本低、原料可再生、绿色环保等优点，为CDs合成提供了新原料^［31］^。植物含有糖、果胶、纤维素、半纤维素、淀粉等物质^［32］^，基于此，相关研究已成功利用菠萝蜜^［33］^、梨汁^［34］^、橘子皮^［35］^等材料实现了CDs的绿色合成。

## 1 **糖衍生**CDs**的合成方法**

自CDs被发现以来，如何以可控的方式合成CDs一直是科学家们研究的重点。根据碳源不同，CDs的合成策略大致可分为“自上而下”和“自下而上”两种^［36，37］^。“自上而下”的合成方法通过物理或化学的方法从较大的碳源中剥离制备尺寸小的CDs。“自上而下”的碳源一般包括碳纳米管^［5，38］^、石墨^［6］^、活性炭^［39］^等。相反，“自下而上”的合成方法利用分子或离子状态下的小碳源合成CDs。自下而上的碳源主要是有机小分子或低聚物，例如柠檬酸^［40］^、糖^［41］^、聚乙二醇^［42］^和尿素^［43］^等。

糖具有较高的含碳量，在CDs合成中起着碳前体的重要作用，已被用于CDs的合成^［44］^。其主要通过水热法、微波法、超声法、热解法等“自下而上”的合成方法制备CDs。

### 1.1 水热法

水热法是指通过密闭反应容器（如高压反应釜）调控温度和压力参数，利用高温高压与水发生反应的一种合成方法^［45］^。该方法具有绿色环保、成本低、无毒、操作简单等优点，受到众多研究者的广泛使用^［37］^。Yang等^［46］^将氨基葡萄糖溶于去离子水中，在特氟龙高压釜中140 ℃水热反应12 h，透析后冷冻干燥2天，在60 ℃真空干燥，得到在430~470 nm激发光下发射绿色荧光的CDs。氨基葡萄糖衍生的CDs具有较多的亲水性官能团，在水环境中稳定。Shchipunov等^［47］^将几丁质和硝酸溶液混合，在特氟龙高压釜中180 ℃水热反应3 h，过滤离心透析，得到几丁质衍生的CDs。此CDs在紫外激发下呈蓝色发光，具有优异的稳定性。Kim等^［48］^将海洋多糖硫酸软骨素钠溶于去离子水中，在聚丙烯高压釜中240 ℃水热反应3 h，产物依次经过过滤、透析、萃取、离心，得到硫酸软骨素钠衍生的CDs。该CDs在430~440 nm的发光二极管（light emitting diode， LED）光下发射绿色荧光，在365 nm的紫外光下发射浅蓝色荧光，具有多色光致发光（photoluminescence，PL）的特性，且具有低毒性。此外，当用CDs处理斑马鱼幼虫时，在肠道中可以选择性地检测到绿色和蓝色荧光，具有体内成像的潜力。

### 1.2 微波辅助法

微波辅助法是一种在微波辐射下将有机物直接碳化成CDs的常用方法，具有效率高、操作方便、设备简单等优点，在大规模制备荧光CDs具有很大潜力^［49］^。2009年，Zhu等^［49］^首次采用微波辅助法将葡萄糖、聚乙二醇-200与蒸馏水混合，通过500 W微波辅助合成糖源CDs。该CDs具有丰富的表面功能基团、小尺寸效应和一定的导电性，因此具有明亮、稳定的发光性能和优异的水分散性能。自此以后，越来越多微波辅助法合成的糖源CDs被报道。Wang等^［50］^将碳水化合物（甘油、乙二醇、葡萄糖、蔗糖）和CuSO_4_混合，微波照射14 min得到光致发光CDs。与其他方法相比，此方法具有合成时间短，不需要表面钝化试剂的优点。Chowdhury等^［51］^将壳聚糖、甘油和乙酸溶液混合制备壳聚糖水凝胶，取部分壳聚糖水凝胶溶解于乙酸中，微波加热5 min，得到壳聚糖水凝胶基CDs。在pH值为3时CDs具有强烈的蓝色荧光，而在pH值为1或5时CDs具有宽泛的荧光。此外，人们还采用壳聚糖/Ag和壳聚糖/Au纳米复合材料制备CDs，所得CDs具有较广的发光范围，且发光强度明显增强。

### 1.3 超声法

超声法是利用高频机械振动在液相介质中诱发空化反应，促进前驱体分解并驱动化学反应，该法能够产生其他方法无法获得的CDs^［52，53］^。Ma等^［54］^将葡萄糖加入到氨水和去离子水的混合溶液中，室温下300 W超声反应24 h，透析得到NCDs。NCDs具有良好的水分散性，无需任何表面修饰就能具有稳定的强可见光发射和优异的上转换PL。在可见光下，制备的NCDs对甲基橙的降解表现出优异的光催化性能。Qiang等^［55］^将马铃薯淀粉、盐酸溶液和水混合，400 W超声反应6 h，然后90 ℃ 加热6 h，过滤透析，得到绿色发光的CQDs。所制备的CQDs具有高水溶性、pH敏感性和离子强度依赖性的特点。此外，CQDs可以作为一种高选择性和灵敏度的荧光探针，以较低的检出限检测水溶液中的Zn^2+^。

### 1.4 热解法

热解法是一种在高温和可控压力下进行的不可逆反应，通常引发有机材料的物理和化学变化，最终生成含有碳的固体残留物。该方法具有操作简单、耗时短、成本低、可大规模生产等优点^［56］^。Stan等^［57］^将木质纤维素废料干燥、细磨后分散于水中，热解处理制备了具有强蓝光发光的CDs。所制备的CDs在438~473 nm的激发波长范围内具有特征波长依赖性，在350 nm激发波长处的量子产率达28%。Zheng等^［58］^将D-葡萄糖、L-天冬氨酸（aspartic acid，Asp）与NaOH水溶液混合，在200 ℃条件下热解20 min，得到CD-Asp。合成的CD-Asp具有较高的生物相容性和可调谐的全彩发光性能。此外，CD-Asp对C6胶质瘤细胞具有较高的选择性和靶向性，具有脑胶质瘤靶向荧光显像剂的潜力。

## 2 以糖为碳源CDs的应用

糖衍生CDs具有独特的光学特性、比表面积大、尺寸小、可调谐发光、低细胞毒性、生物相容性和光稳定性的优点，成为生物医药和环境分析应用的理想选择^［59，60］^。通过各种技术制备的糖源CDs已成功在多个领域应用，包括生物成像、生物传感、药物/基因载体、色谱分析等。

### 2.1 生物成像

在生物成像技术中，需要X射线、超声和磁共振成像等技术辅助完成^［61］^。CDs具有低毒性、生物相容性和优异的荧光特性等优点，更有利于生物系统的体外和体内可视化，是生物成像的优秀候选者^［62］^。

Yang等^［63］^以葡萄糖为碳源，与KH_2_PO_4_水热反应得到单分散、光稳定的绿色荧光CDs。该CDs的荧光发射可以通过改变KH_2_PO_4_的浓度来调节。此外，细胞摄取研究表明，葡萄糖衍生CDs可以作为生物成像剂标记HepG2细胞。并通过4′，6-二脒基-2-苯基吲哚（4′，6-diamidino-2-phenylindole，DAPI）的反染色研究，确定了CDs是在细胞核周围的细胞质中进行定位。Zhang等^［64］^以透明质酸（hyaluronic acid，HA）为碳源，与甘氨酸水热碳化得到透明质酸衍生HA-CDs（图1）。该HA-CDs具有激发依赖性，在紫外激发下发射蓝色荧光，在496 nm激发下发射绿色荧光。HA-CDs表现出优异的荧光特性、良好的胶体稳定性和良好的生物相容性，因此易于进入过表达CD44的癌细胞细胞质，特别是在细胞核周围。因此，HA-CDs在肿瘤靶向成像和标记中可以作为CD44高表达的新型细胞特异性荧光探针。

**图1:**
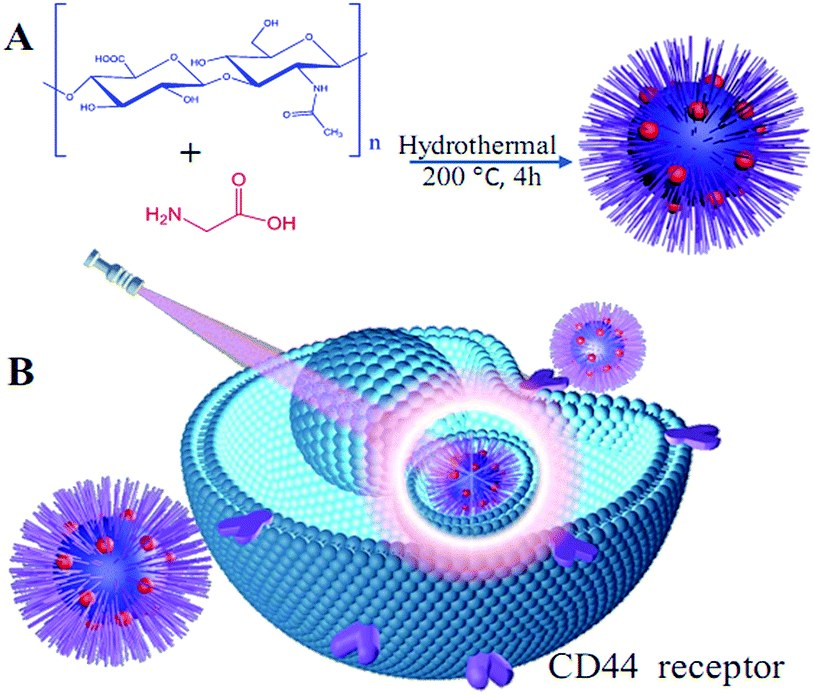
HA-CDs的（A）合成及（B）生物成像应用^［64］^

CDs不仅可以依赖自身的发光特性在体内成像，还可以与其他技术结合，使其适用于体内可视化检测。Yang等^［65］^采用葡萄糖制备了CDs。所制备的CDs表现出强烈的荧光、优异的稳定性和低毒性。CDs与荧光Zn^2+^探针槲皮素（QCT-Zn^2+^）结合，建立了基于荧光共振能量转移（fluorescence resonance energy transfer，FRET）系统的CDs/QCT-Zn^2+^荧光探针，对Zn^2+^具有良好的灵敏度和选择性，线性范围为2~100 μmol/L，检出限达到2 μmol/L。此外，该方法还能用于对Hela细胞中Zn^2+^的分布进行成像。

### 2.2 生物传感

CDs作为生物传感探针的适用性源于其可调的表面官能团、高电子转移效率和优异的电化学性能，这些特性赋予材料优异的环境适应性和灵敏度^［66］^。此外，CDs具有体积小，灵敏度高等优点，可使用较少的样本检测目标物质，降低成本^［67］^。CDs应用的生物传感器包括生物材料传感、离子传感、pH传感和温度传感等。

#### 2.2.1 生物材料传感

CDs具有独特的特性，如激发依赖性发光、更高的光稳定性、低的细胞毒性和更好的水溶性等，在生物材料传感应用中具有很大的潜力^［68］^。

抗生素是常用的抗菌剂^［69］^，广泛用于水产或家畜养殖系统^［70］^和人类医疗等领域^［71］^。抗生素的滥用会对环境和人类健康产生巨大威胁^［72］^。因此，利用糖源CDs构建的传感器为检测抗生素提供了一种成本低、操作简便的新方法，部分成功应用于实际复杂样品中抗生素的检测。Yang等^［73］^以葡萄糖为碳源，与盐酸回流合成了CDs。该CDs具有化学稳定性和独特的荧光特性，成功用于检测诺氟沙星（norfloxacin，NOR），线性范围为2.00×10^-5^~1.33×10^-8^ mol/L，检出限为1.33×10^-8^ mol/L。Dinç等^［74］^以生物质蔗糖为碳源，首次不使用任何化学或热处理的方法制备了强发光CDs。该CDs对四环素（tetracycline，TC）具有高选择性，且荧光强度与四环素浓度成线性（*R* ^2^=0.995 2），可用于TC定量检测。Zhao等^［75］^以废弃的二乙酸纤维素为碳源，与氢氧化铵水热反应得到N-CDs。所制备的N-CDs具有优异的荧光性能，对TC表现出高选择性，在0~80 μmol/L浓度范围内，荧光猝灭强度与TC呈良好的线性关系，检出限为0.06 μmol/L。Guo等^［76］^以红糖为碳源，通过一步水热法合成了CQDs。红糖衍生的CQDs具有良好的水分散性和稳定的荧光特性。CQDs的荧光会被金纳米颗粒（AuNPs）有效猝灭，庆大霉素和CQDs共同竞争AuNPs的结合，最终导致AuNPs在溶液中聚集，释放CQDs恢复其荧光。因此，通过检测CQDs荧光强度的变化可以实现庆大霉素的选择性检测和定量。此外，在西瓜汁和黄瓜汁中，该CQDs对庆大霉素的检测线性范围为0.56~5.56 μmol/L和0~555.56 nmol/L。该方法具有线性范围宽、检出限低、操作简单、成本低等优点，且成功应用于食品样品中庆大霉素的检测。

此外，糖源CDs还用于其他常见生物材料的检测。Chen等^［77］^以乳糖为碳源，与NaOH溶液简单加热合成了CDs。叶酸官能团（-OH，-COOH和-NH_2_）与CDs的-OH和-COOH之间形成氢键作用，导致CDs荧光猝灭，因此该CDs实现了叶酸的选择性检测。CDs对叶酸检测的线性范围为6×10^-5^~8×10^-8^ mol/L，检出限为1.2×10^-9^ mol/L。此外，该CDs成功实现了人体尿液样本中叶酸的测定，验证了该方法的实用性。Zhang等^［78］^以葡萄糖为碳源，聚乙烯亚胺（polyethyleneimine，PEI）为氮源，水热法合成了PEI-CDs。由于Cu^2+^会有效猝灭PEI-CDs的荧光，开发了具有优异的光学稳定性和良好的生物相容性的PEI-CDs-Cu^2+^体系，实现了谷胱甘肽（glutathione，GSH）的选择性检测。此外，所开发的体系已成功应用于MGC-803细胞中GSH的检测。

#### 2.2.2 离子传感

离子检测主要采用荧光分析法，通过监测目标离子与CDs相互作用引发的PL强度变化实现检测。CDs的PL强度在较宽的离子浓度范围内表现出良好的线性，且检出限低，可在10^-7^或更低的离子浓度下实现精准检测^［79］^。目前，大多数糖衍生CDs主要应用于金属离子检测，且成功实现了在水样、血清等一些复杂样品中的检测，为人类的生活和健康提供了保障。

Shi等^［80］^以葡萄糖为碳源，与氨和磷酸一锅水热法得到N，P-CDs。N，P-CDs对Fe^3+^具有高灵敏度和选择性，检出限低至1.8 nmol/L。同时，在复杂生物样品中，Fe^3+^的监测结果和细胞内Fe^3+^的荧光图像表明，N，P-CDs具有低细胞毒性和良好的生物相容性，有望作为临床诊断的有效探针。Wang等^［81］^以葡萄糖为碳源，与硼酸水热反应得到B-CDs。所制备的B-CDs显示出稳定的蓝色荧光，在水中具有良好的分散性。B-CDs对Fe^3+^离子具有良好的选择性和灵敏度，检出限为242 nmol/L，线性范围为0~16 μmol/L。此外，B-CDs已经成功应用于人工水样中Fe^3+^的检测。Wang等^［82］^为了获得更亮的CDs，以麦芽糖为碳源，与磷酸和盐酸水热处理制备了P，Cl-CDs。P，Cl-CDs具有很强的光稳定性，荧光被Fe^3+^强烈猝灭。在0.1~8.0 μmol/L范围内，P，Cl-CDs的荧光随Fe^3+^浓度的增加而降低，检出限为60 nmol/L，乙二胺四乙酸（ethylene diamine tetraacetic acid，EDTA）可以使P，Cl-CDs的荧光恢复。因此，P，Cl-CDs被用作检测Fe^3+^的高选择性荧光纳米传感器。此外，P，Cl-CDs实现了加标血清和水样中Fe^3+^的检测。Kolekar等^［83］^以棕榈糖为碳源，与硫酸一步水热法制备了蓝色荧光的S-CDs。S-CDs具有毒性低、水溶性好、抗干扰性好、荧光稳定等特点。S-CDs特异性识别Cr^6+^和Fe^3+^，检出限分别为4.25 mg/mL和3.15 mg/mL（图2）。此外，S-CDs可实现实际水样中Cr^6+^和Fe^3+^离子的检测，并具有良好的回收率。随后，该课题组^［84］^为了提高检测的灵敏度，以棕榈糖为碳源，与硫酸直接加热制备了硫掺杂DCDs。DCDs在360 nm激发波长和460 nm发射波长下具有亮蓝色荧光。DCDs选择性检测Fe^3+^，检出限为1.92 μg/mL，定量限为5.84 μg/mL。

**图2:**
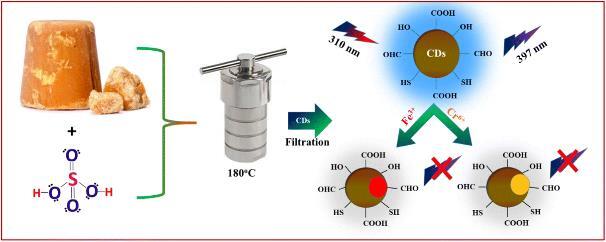
S-CDs的合成及其在Cr^6+^和Fe^3+^检测中的应用^［83］^

糖衍生的CDs除了常用于Fe^3+^的检测，对环境中其他常见的金属离子同样具有良好的检测能力。Rahmani等^［85］^以黄芪胶为碳源，与乙二胺水热反应得到N-CDs。N-CDs具有较高的荧光强度、较低的细胞毒性、良好的生物相容性和抗氧化活性。N-CDs对Au^3+^具有较高的灵敏度和选择性，在较宽的线性范围内检测Au^3+^。Jayaweera等^［86］^以生物质纤维素为碳源，与尿素和硝酸铝水热碳化得到N/Al-CDs。N/Al-CDs具有抗光漂白性和高度光稳定性的特点。此外，N/Al-CDs对Mn^7+^具有高选择性和高灵敏度，线性范围为0~100 μmol/L，检出限为46.8 nmol/L。此外，N/Al-CDs可对实际水样中的Mn^7+^进行定量检测。

近几年，核污水的排放对人类的生活造成了巨大的威胁^［87］^，CDs在核素检测方面展现出了巨大的潜力。Mahmoud等^［88］^以淀粉为碳源，采用微波辅助法得到CQDs。CQDs与聚合物基质载体（PAFP）制备了新型纳米生物吸附剂CQDs@PAFP。所制备的CQDs@PAFP是一种吸附废水和海水中核素U^6+^的优良纳米生物吸附剂，吸附率分别为97.3%和96.0%，且可重复使用。因此，此吸附系统的发明可以简单、高效、低成本地从水中提取U^6+^，对环境和人类健康发挥重要作用。

#### 2.2.3 pH传感器

CDs表面的官能团在不同pH环境中发生质子化或去质子化反应，改变表面的电子状态和电子结构，可以影响荧光特性，从而实现pH传感^［89］^。CDs的pH传感特性可在癌症等严重疾病的体内早期诊断^［90］^和环境水样的监测方面发挥重要作用^［91］^。

Liu等^［92］^以葡萄糖为碳源，与食用油混合加热制备了H-CDs。H-CDs具有优异的光学性能、低细胞毒性、良好的生物相容性和光稳定性。此外，H-CDs对pH值有良好的荧光响应，线性范围在3.0~13.0，且已成功应用于环境样品和Hela细胞的pH监测（图3）。Barati等^［93］^以葡萄糖为碳源，采用一锅水热法合成了NCDs。NCDs对pH的荧光变化具有强烈的激发依赖性。利用这一特性，在酸性（pH 2.0~8.0）和碱性（pH 7.0~14.0）pH范围内的发射光谱内构建了两个独立的比率荧光式pH传感器。使用合适的多元校准方法，该pH传感器可有效校准2.0~14.0的pH范围，平均预测误差低至0.067 pH单位，并具有很好的稳定性。此外，NCDs已成功应用于实际水样中pH值的测定，具有良好的准确度和重复性。

**图3:**
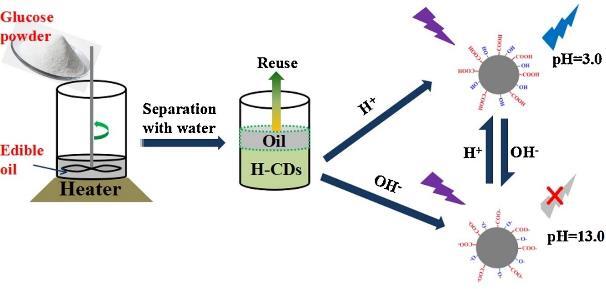
H-CDs的制备及pH响应^［92］^

#### 2.2.4 多功能传感

最近，生物学的进步使CDs实现多功能应用，能够检测各种类型的化合物。例如，这些CDs可以同时用于pH值和离子传感以及成像等。这一特性使它们在不久的将来有望用于体内监测。

Karami等^［94］^以葡萄糖为碳源，与硫酸和3-硝基苯胺水热处理，制备了新型的本征双发射CDs。当pH值在4.0~5.0范围内时，在400 nm和610 nm处显示出双发射峰。CDs在400 nm的第一个发射峰能被Cu^2+^选择性淬灭，线性范围为0.01~1.00 mmol/L，检出限为7.0 nmol/L。此外，在pH为4.0时，Asp能够恢复CDs-Cu^2+^体系的猝灭荧光。因此，CDs可通过比率荧光方法选择性测定Asp，线性范围为0.2~15 mmol/L。该CDs已经成功实现了河流水样中Cu^2+^和人血清样品中Asp的检测。Liao等^［95］^以纤维素二糖为碳源，采用水热法合成了N-CDs。N-CDs的荧光对温度变化具有线性响应。在10~80 ℃温度范围内，制备的N-CDs具有可逆和可恢复的荧光特性。此外，所制备的NCDs在230 nm和320 nm激发处具有双荧光中心，在207 nm和270 nm处具有双吸收带，可用于新型的双模式比率测定法检测伏杀磷；基于双荧光中心比率测定法的线性范围为0.12~5.45 μg/mL，检出限为42.90 ng/mL；而基于双吸收的比率测定法的线性范围和检出限分别为0.02~1.40 μg/mL和6.67 ng/mL。

### 2.3 药物/基因载体

CDs具有易于表面功能化、体积小、毒性低、生物相容性高等优点，可作为药物/基因传递的载体。由于CDs固有的荧光特性，能够追踪药物/基因传递的途径，是其他荧光染料或半导体纳米颗粒的优秀替代品^［96］^。

Dubey等^［97］^将D-（+）-葡萄糖与磷酸混合，采用微波辅助法制备了CDs。阿霉素（doxorubicin，Dox）通过形成酸不稳定的共价和非共价相互作用与CDs偶联，得到CDs-Dox-ADH自组装系统。CDs-Dox-ADH在酸性pH下水解并释放Dox，显著增强了CDs-Dox-ADH在宫颈癌细胞中的细胞毒性。与游离的DOX相比，CDs-Dox-ADH在癌细胞中的毒性更高，在正常细胞中的毒性低。Chung等^［98］^将氨基葡萄糖与壳聚糖-聚乙二醇聚合物（CP）和氨混合，采用微波辅助法制备CD-CP，并制备了基于氧化铁和CD-CP的纳米粒子（CNPCP）。所制备的CNPCP在水环境中单分散，并显示激发依赖性荧光，且在生物培养基中表现出良好的尺寸稳定性和荧光强度。CNPCP具有毒性低、快速定量成像的优点，可作为肿瘤细胞系荧光探针。此外，将DOX与CNPCP偶联（CNPCP-DOX），能够以剂量依赖的方式杀死癌细胞。因此，CNPCP作为荧光探针具有递送化疗药物的潜力。Gogoi等^［99］^将壳聚糖衍生CDs^［51］^作为钙藻酸盐（calcium alginate，CA）微珠的保护层（CD-CA），并将TC负载到CD-CA微珠表面。与未包被微珠相比，其载药量增加了两倍。此外，以*β*-环糊精/四环素（*β*-TC）主客体包合的复合物在CD-CA微珠上具有更高的负载水平，且每个pH值下的TC释放速度较慢，pH为1时具有最大的释放量，可应用于pH较低的胃肠道。因此，壳聚糖水凝胶基CDs具有pH值响应型给药载体的应用潜力。

Chen等^［100］^以紫菜多糖为碳源，以乙二胺作为表面钝化剂，水热碳化得到紫菜多糖衍生的CDs。所制备的CDs具有高量子产率（56.3%）、激发依赖性荧光、表面带正电荷、低细胞毒性和卓越的凝聚大分子质粒DNA的优点。CDs首次被用于外胚层间充质干细胞的神经元诱导分化。因此，该CDs可作为一种新的非病毒基因载体用于成体干细胞的神经分化，在组织工程和生物成像方面具有很大的前景。Zhou等^［101］^以海藻酸钠为碳源，与过氧化氢水热碳化得到海藻酸钠衍生的CDs。该CDs具有凝集质粒DNA的能力、良好的生物相容性、低毒性和优异的上转换特性等显著性能，可作为优良的DNA凝聚剂。该CDs能追踪自身进入细胞的路径，且还能追踪CDs/pDNA复合物的内吞机制，因此具有基因载体和监测基因转移的生物成像探针的双重作用，在生物医学应用中具有巨大的潜力。

### 2.4 色谱分析

色谱技术的发展主要依赖于色谱固定相的制备和新的检测方法，而固定相是色谱柱的核心，直接影响化合物的分离，因此研究人员开发了一系列新型色谱固定相，以提高其分离性能^［102］^。其中，CDs表面具有丰富的亲水基团和疏水基团、体积小、在硅胶表面分布均匀、表面的活性位点多等特点，可与多孔硅胶结合制备新的色谱固定相，用于提高各种化合物的分离性能。

Li等^［103］^以废弃纤维素烟尘为碳源，与硝酸回流得到CNPs。并选择3-（氨基丙基）三甲氧基硅烷（3-aminopropyltrimethoxysilane，APTMS）作为偶联剂，首次将CNPs接枝到硅胶表面，以制备新型固定相。该固定相在亲水相互作用色谱（hydrophilic interaction chromatography，HILIC）和全水液相色谱（per aqueous liquid chromatography，PALC）模式下对4种磺胺类化合物、5种核苷类化合物实现基线分离。5种核苷可在15 min内分离，胸腺嘧啶的柱效可达32 990 N/m。此外，该色谱柱已经成功分离红花注射液中的12个化合物。Yuan等^［104］^制备了一种基于葡萄糖衍生CDs-二氧化硅修饰的新型固定相Sil-Glc-CDs（图4）。Sil-Glc-CDs色谱柱对极性分析物具有更好的保留能力和分离选择性，包括氨基酸、糖苷、人参皂苷、抗生素、核苷和核碱基。其中，5种氨基酸可在10 min内分离，鸟苷的柱效高达43 800 N/m。且该色谱柱具有良好的稳定性和批次间重现性，相对标准偏差（relative standard deviation，RSD）为0.80%~1.97%（*n*=3）。此外还成功地将该固定相用于枸杞水溶液中葡萄糖和果糖的定量，质量浓度分别为2.2 mg/mL和3.4 mg/mL。随后，该课题组^［105］^合成了一种新型的葡萄糖基N掺杂的CDs（Glc-NCDs），并在硅胶表面修饰，制备了Sil-Glc-NCDs固定相。由于N掺杂CDs官能团之间的协同作用，Sil-Glc-NCDs色谱柱比之前的Sil-Glc-CDs色谱柱具有更强的分离选择性，可用于碱基、核苷、抗生素等的分离。其中，8种人参皂苷均可在10 min内分离，且色氨酸峰的柱效高达68 000 N/m。此外，该固定相具有良好的稳定性（RSD=0.32%~0.97%，*n*=10）和重现性。该固定相已经成功测定了胶囊中罗红霉素的含量，质量浓度为2.45 mg/mL。Chen等^［106］^合成了十八胺和葡萄糖衍生的疏水CDs（Glc-OCDs），并采用“Nano-on-Micro”策略将其接枝到多孔二氧化硅表面，制备了反相液相色谱的新型固定相Sil-Glc-OCDs。该固定相能够很好地分离7种多环芳烃、8种烷基苯、8种酚类和7种磺胺类化合物，且在分离叔丁基苯、仲丁基苯、异丁基苯和正丁基苯的异构体中表现出良好的色谱选择性。此外，还能对黄芪提取物定量，其中毛蕊异黄酮苷、芒柄花素、毛蕊异黄酮、刺芒柄花素、染料木素和异鼠李素质量浓度分别为0.15、0.088、0.14、0.086、0.18和0.29 g/L。

**图4:**
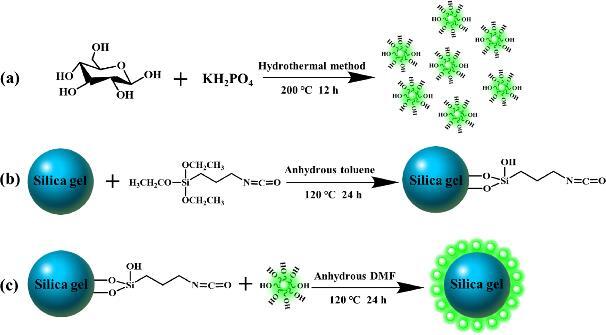
（a）Glc-CDs和（b，c）Sil-Glc-CDs材料的合成^［104］^

最近，我们课题组将CDs分别与分子印迹技术相结合，制备了适用于核苷类、磺胺类及其他类抗生素分离的CDs掺杂分子印迹聚合物^［107］^。为了进一步提升固定相对核苷类、生物碱类等强极性药物及含氟农药的分离性能，本课题组设计、合成了CDs掺杂含氟色谱固定相（原理如图5所示）^［108］^。

**图5:**
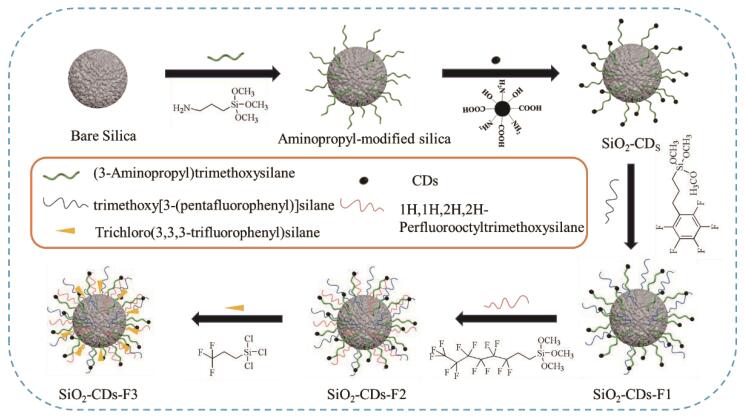
F3-CDs-SiO_2_ 固定相的制备原理^［108］^

## 3 总结

CDs具有优异的水溶性、化学稳定性、低毒性、良好的生物相容性、低成本、环境友好性和独特的光学特性，已在生物医学和生物化学领域展现出巨大的应用潜力。糖类化合物是自然界含量最丰富的碳水化合物，由于其具有低毒性、广泛的生物活性、低渗透效应等独特的性质，在CDs的合成中发挥重要作用。本文综述了以糖作为廉价和容易获得的起始材料制备CDs的合成方法，以及结合生物成像、生物传感、药物/基因载体、色谱分析等手段在药物分析领域的应用。尽管人们为糖源CDs的开发做出了诸多努力，但仍存在许多挑战和阻碍。首先，CDs的光致发光机制尚未完全了解。为了扩大糖源CDs的应用，迫切需要一种详细的CDs光致发光机制。其次，糖源CDs的未来展望和研究，包括开发具有成本效益的大规模生产、物理和化学稳定性、简便的制备程序、表面官能团修饰、各种杂原子掺杂以及提高量子产率等，这些挑战将成为未来研究的重要目标。最后，还需要进行系统的研究，以合成无毒、生物相容性好的糖源CDs，使其能够广泛应用于药物/基因递送、生物成像、生物传感等生物医药领域。
